# Machine learning to predict gold nanostar optical properties†

**DOI:** 10.1039/d5na00265f

**Published:** 2025-05-27

**Authors:** Peiying Wu, Rui Zhang, Céline Porte, Fabian Kiessling, Twan Lammers, Sima Rezvantalab, Sara Mihandoost, Roger M. Pallares

**Affiliations:** a Institute for Experimental Molecular Imaging, RWTH Aachen University Hospital Aachen 52074 Germany rmoltopallar@ukaachen.de; b Fraunhofer Institute for Digital Medicine MEVIS Bremen 28359 Germany; c Chemical Engineering Department, Urmia University of Technology Urmia 57166-419 Iran s.rezvantalab@uut.ac.ir; d Electrical Engineering Department, Urmia University of Technology Urmia 57166-419 Iran s.mihandoost@uut.ac.ir

## Abstract

Gold nanostars (AuNS) are nanoparticles with spiky structures and morphology-dependent optical features. These include strong extinction coefficients in the visible and near-infrared regions of the spectrum, which are commonly exploited for biomedical imaging and therapy. AuNS can be obtained *via* seedless protocols with Good's buffers, which are beneficial because of their simplicity and the use of biocompatible reagents. However, AuNS growth and optical properties are affected by various experimental factors during their seedless synthesis, which affects their performance in diagnosis and therapy. In this study, we develop a workflow based on machine learning models to predict AuNS optical properties. This approach includes data collection, feature selection, data generation, and model selection, resulting in predictions of the first and second localized surface plasmon resonance positions within 9 and 15% of their true values (root-mean-squared percentage error), respectively. Our results highlight the benefits of using machine learning models to infer the optical properties of AuNS from their synthesis conditions, potentially improving nanoparticle design and production for better disease diagnosis and therapy.

## Abstract 2

Machine learning can infer the optical properties of gold nanostars from their synthesis conditions.

## Graphical abstract

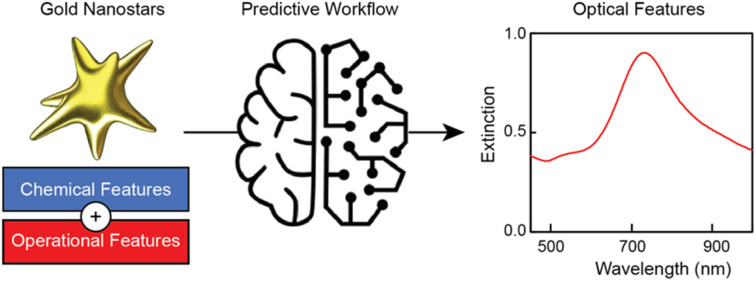

## 1. Introduction

Gold nanostars (AuNS) are anisotropic gold nanoparticles with star-shaped morphology, including sharp and pointed tips radiating from a central core.^1^ AuNS exhibit morphology-tunable optoelectronic properties, which are highly valued in numerous applications, ranging from biomedicine to catalysis.^2–5^ For instance, AuNS sustain localized surface plasmon (LSP) resonances and intense near-field enhancements, particularly at the sharp end of their branches.^6^ The intense field enhancements are commonly exploited in identifying and quantifying analytes *via* surface-enhanced Raman spectroscopy.^7–9^ At the same time, their sensitivity to changes in the refractive index of their surroundings is used for colorimetric sensing.^10–13^ Because their LSP bands can be shifted towards the near-infrared region of the spectrum by increasing the aspect ratio of their branches, AuNS have been preclinically used as theranostic agents for photothermal therapy and photoacoustic imaging.^14–16^

**Fig. 1:**
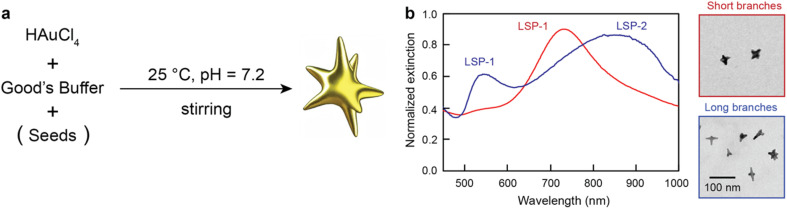
Synthesis of AuNS with Good's buffers. (a) Schematic representation of the synthesis of AuNS with biocompatible Good's Buffers. (b) Representative extinction spectra and transmission electron micrographs of AuNS grown under different conditions and displaying short and long branches and one and two main LSP bands, respectively. The AuNS sample with short branches was synthesized and characterized as described in the methods section. The transmission electron micrograph and UV-vis spectrum of the AuNS sample with long branches has been adapted with permission from ref. 22. Copyright 2022 American Chemical Society.

AuNS are commonly obtained through colloidal chemistry *via* seed-mediated protocols, where gold salts are reduced in the presence of shape-directing agents on top of pre-synthesized spherical gold nanoparticles (used as seeds).^17–19^ Many of these methods, however, tend to rely on cytotoxic and/or strongly bound reagents that can hamper AuNS further use. In the last decade, a seedless methodology based on Good's buffers (Fig. 1a), which act as both reducing and shape-directing agents, has become increasingly adopted for the synthesis of AuNS.^20–22^ This seedless approach (even though it can also be used with pre-synthesized seeds) benefits from (1) being straightforward, *i.e.* a one-pot protocol rather than a multi-step procedure like the seed-mediated syntheses, (2) using biocompatible Good's buffers, which are frequently used as buffers in cell cultures, and (3) providing great tunability over LSP position and number (with some AuNS displaying two main LSP bands) (Fig. 1b). The simplicity of the seedless synthesis, however, comes at a cost: the influence of multiple factors (some of them through secondary effects) on the growth and final morphology and optical properties of AuNS.^22^ As a result, it is difficult to fully understand the role and magnitude of these multifactorial influences on the AuNS optical features, which directly affect their performance in diagnosis and therapy. Hence, approaches that can cope with the large number of variables and the multidimensional relationships between them are needed to assess and predict these multifactorial influences over AuNS optical properties.

In recent years, machine learning (ML) has become a key tool in nanoscience to analyze large datasets of nanomaterials, to identify complex relationships between processes, structures and properties, and to predict nanomaterial features and performances.^23–25^ For example, deep neural networks have been used to predict the structure of spherical gold nanoparticles based on their optical characteristics,^26^ while supervised ML algorithms have been employed to predict the UV-vis spectra of spherical and rod-shaped gold nanoparticles based on their morphology.^27^ Zhang *et al.* explored several ML algorithms to predict the photothermal performance of silica-coated gold nanorods for photothermal therapy, with extreme gradient boosting (XGB) showing the highest prediction accuracy (91%).^28^ It is worth noting that the quality of ML prediction heavily depends on the size of the input data used for training. Hence, multiple training data augmentation approaches, such as those obtained *via* generative adversarial networks (GAN), have been developed to overcome the challenges related to data scarcity.^29–31^ In the case of AuNS obtained through Good's buffer synthesis, we expect that ML can elucidate how the different synthesis parameters affect the optical properties of the resulting particles. This could lead to a more efficient synthetic process, allowing for the development of tailored AuNS with enhanced optical features for specific applications in diagnosis and therapy.

**Fig. 2:**
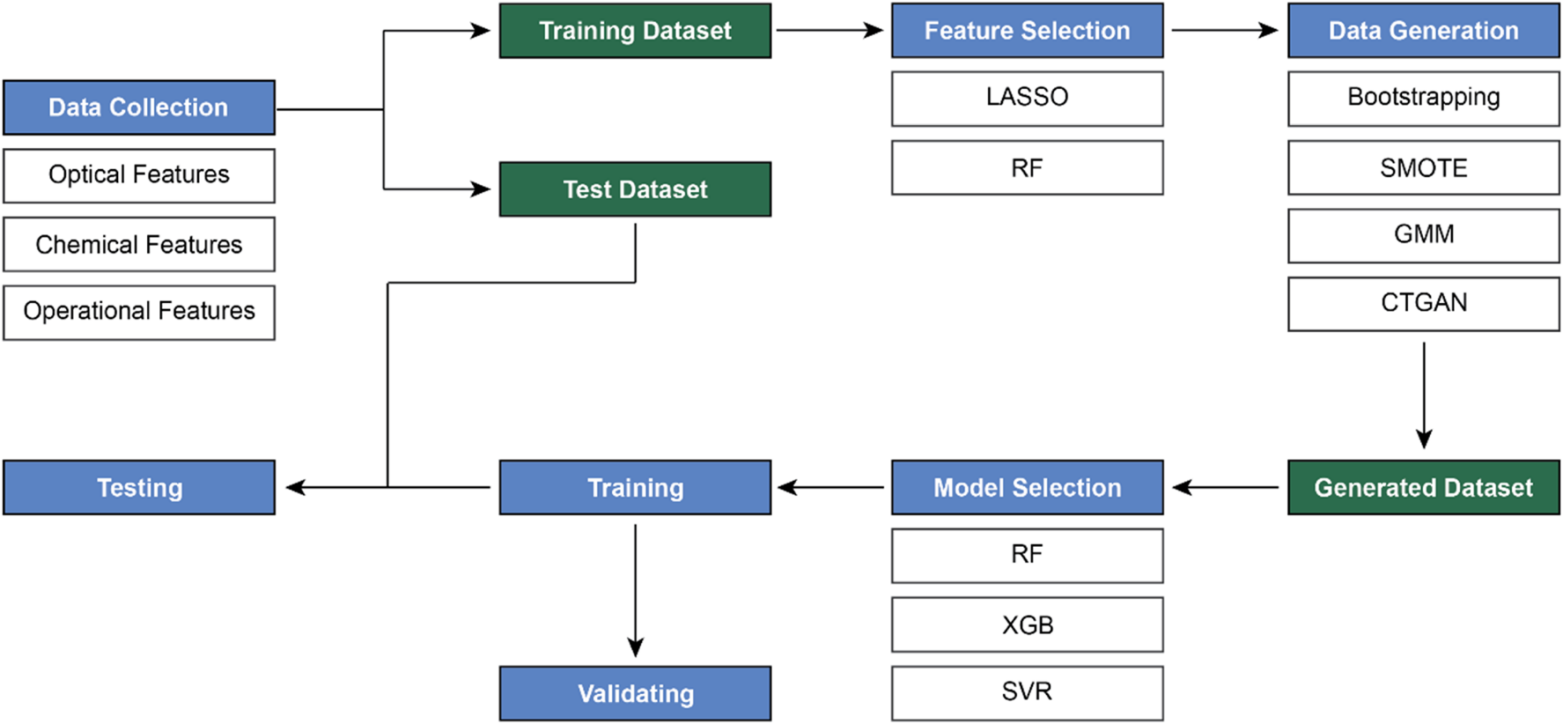
The workflow of the study. Data collection was performed to include features affecting the LSP of AuNS. Feature evaluation and selection were conducted to identify key variables. Data generation techniques were applied to enhance model performance. Model selection was then carried out, followed by the prediction of LSP-1 and LSP-2 values using the most suitable models.

In this study, we developed a comprehensive workflow for predicting the LSP band positions of AuNS using ML models. The process involved data collection, feature selection, data generation, and model selection (Fig. 2). Firstly, the relationships between key features were explored, and feature importance was determined using the least absolute shrinkage and selection operator (LASSO) and random forest (RF) ranking methods, where the former performed more efficiently for feature selection. Moreover, the model performance was optimized with synthetic data generation methods, including bootstrapping, synthetic minority over-sampling technique (SMOTE), Gaussian mixture model (GMM), and conditional tabular generative adversarial network (CTGAN), which significantly improved the accuracy of the ML models, particularly with bootstrapping. Among the different models evaluated, which included RF, XGB, and support vector regression (SVR), the RF model demonstrated the best predictive accuracy. Additionally, we also explored a multi-output RF model to simultaneously predict the position of both LSP bands (LSP-1 and LSP-2).

## 2. Methods

### 2.1. Data collection

Data from AuNS synthesized with Good's buffers were collected from previously published research and our laboratory findings. The dataset comprised 144 samples with 11 features that described the chemical characteristics, solution parameters, and operation conditions (Table 1). We focused on predicting the two LSP band positions (LSP-1 and LSP-2), as they are the most important parameters for most AuNS applications. Moreover, we only considered features with at least 50 data points within our dataset. This threshold was chosen to avoid unreliable predictions, resulting from low-number data points. Hence, the dataset contained several input features, including buffer type (*i.e.* 4-(2-hydroxyethyl)piperazine-1-ethanesulfonic acid (HEPES), 4-(2-hydroxyethyl)-1-piperazinepropanesulfonic acid (EPPS), and 3-(*N*-morpholino)propanesulfonic acid (MOPS), Fig. S1†), buffer concentration (mM), gold concentration (mM), ratio of buffer/gold concentration, pH, seed size (nm), seed concentration (pM), stirring (yes/no), and temperature (°C), and two target outputs, namely LSP-1 (nm) and LSP-2 (nm) (Fig. S2†), which represented the multi-output regression targets.

**Table 1:** List of features considered in the study

	Name	Value ± standard deviation
Target	LSP-1 (nm)	682 ± 79
	LSP-2 (nm)	230 ± 436
Chemical features	Gold concentration (mM)	0.23 ± 0.10
	Buffer type	EPPS, HEPES, MOPS
	Buffer concentration (mM)	109.8 ± 86.0
	Ratio of buffer/gold concentration	522 ± 433
	pH	7.11 ± 0.47
	Seed particle size (nm)	15 ± 25
	Seed concentration (pM)	3.2 ± 6.1
Operation features	Temperature (°C)	25, 50
	Stirring	Yes/no

### 2.2. Feature initial evaluation and selection

Feature evaluation and selection were conducted to identify the most relevant descriptors for predicting AuNS LSP-1 and LSP-2. Initially, a correlation analysis was performed on the original dataset to evaluate the relationships between the features and detect any potential redundancies or multicollinearity. A correlation matrix and pair plots were used to visualize interactions among the features, which guided the selection of the most relevant variables for further analysis.

After this initial evaluation, the importance of the features was assessed using RF and LASSO. For RF, the Gini importance metric, which evaluated the reduction in impurity achieved by each feature, was used to assess the importance of each feature. For LASSO, a penalty coefficient (*λ*) was conducted systematically using LassoCV, which performed cross-validation to identify the optimal *α* (inverse of *λ*) from a specified range of values. The *λ* was determined through 5-fold cross-validation by minimizing the mean squared error across a grid of 50 *α* values logarithmically spaced between 10^−3^ and 10^1^. After fitting the model, feature importance was assessed based on the absolute values of the LASSO coefficients. These methods allowed for a detailed ranking of the features based on their contribution to the prediction of the LSP values. The RF algorithm was employed for its ability to capture complex non-linear relationships between features, while LASSO was selected for its efficiency in handling high-dimensional data by penalizing less relevant features and shrinking their coefficients to zero.^32,33^

### 2.3. Data generation

To train the prediction models effectively and enhance the original data, we compared the performance of synthetic data generation methods, including bootstrapping, SMOTE, GMM, and CTGAN. Based on our comparison, we selected bootstrapping, which generates additional samples by random sampling with replacements from the original dataset.^34^ This technique was applied to increase the size of the training data and improve the models' ability to generalize. Bootstrapping ensured that the original distribution of the data was preserved, while providing a larger dataset to train the models. SMOTE addressed any imbalances in the dataset, particularly if some features were underrepresented. SMOTE works by creating synthetic samples from the minority class by interpolating between existing samples.^35^ This method helped balance the dataset and prevented the models from being biased toward more frequent feature values. GMM was used to generate synthetic data based on the underlying statistical distribution of the original dataset.^36^ By fitting a GMM to the dataset, the method generated new samples that retained the statistical characteristics of the original dataset. GMM is particularly effective in capturing complex patterns and distributions in the input data, helping to improve the overall diversity and coverage of the feature space. CTGAN is a variation of GAN that is tailored to generate realistic tabular data.^37^ It introduces conditional sampling to better handle discrete columns and data imbalance. It is applied to the dataset to create highly realistic synthetic data by learning from both continuous and categorical variables. CTGAN captures the joint distributions between features, producing synthetic data that closely mimics the structure of the original dataset, further enriching the training data. By fitting different methods to the original dataset, we generated the same number of samples as the original data (144). This synthetic approach effectively doubled the size of the training set, ensuring a greater representation of the feature space. The generated synthetic data were combined with the original data, resulting in an augmented dataset to train the ML models.

### 2.4. LSP prediction

#### 2.4.1. Regression algorithms

To predict the position of the LSP-1 and LSP-2 bands, we experimented with three regression algorithms, namely RF, SVR, and XGB. For RF, the optimal hyperparameters were determined through grid search, with 100 estimators and a random state of 42. This model was selected for its ability to handle non-linear interactions and complex feature dependencies. A non-linear radial basis function kernel was selected for SVR, ensuring a direct relationship between input features and the target variables. The gamma parameter was set to ‘scale’ to control the influence of individual data points. The XGB model was optimized using key hyperparameters, including n_estimators = 50, learning_rate = 0.2, max_depth = 3, colsample_bytree = 0.8, and subsample = 0.8. The XGB was selected for its ability to ensure a balance between model flexibility and performance.

#### 2.4.2. Multi-output regressor

To predict LSP-1 and LSP-2 simultaneously, we used the XGB model, as it was the model with the best performance in single output prediction. We employed synthetic data generation to address the potential issue of limited data.

### 2.5. Model evaluation

#### 2.5.1. Metrics

To evaluate the performance of the regression models, we used three different metrics, namely mean square error (MSE), mean absolute error (MAE), and *R*-squared (*R*^2^). MSE measures the average squared difference between the predicted and actual values.
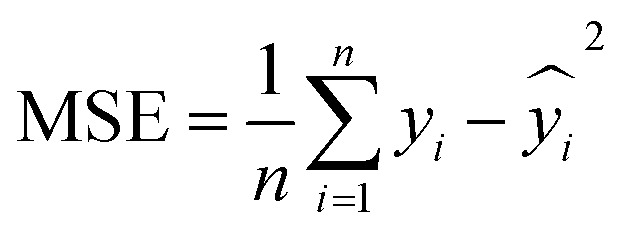


MAE captures the average magnitude of errors in the predictions.
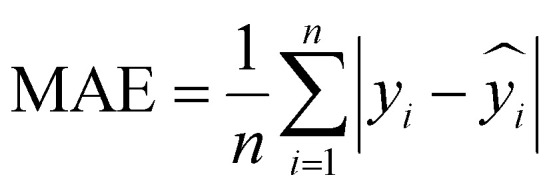


*R* ^2^ assesses how well the model explains the variability in the target data.
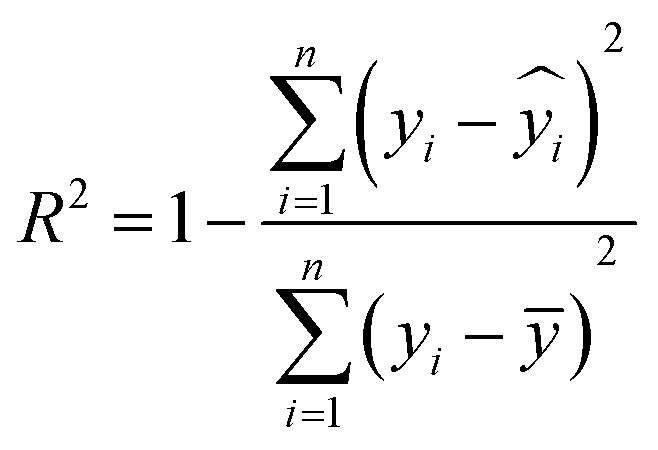


#### 2.5.2. Evaluation method

The whole data was divided into training (80%) and test (20%) sets to ensure that the test data was untouched. Next, the method validation was conducted using K-fold cross-validation (with 7-fold). After normalizing the training set with StandardScaler to maintain uniform scaling of the feature values, synthetic data were generated exclusively for the training set. The training data, including both the original and synthetic samples, were split into seven-fold, where six-fold were used for training, and one-fold was held out for validation. This process was repeated seven times, allowing each fold to be used as the validation set once. During each iteration, the model was trained and validated, and the *R*^2^, MAE, and MSE scores were recorded for both the training and validation sets. To provide further insights into the models' performances, visualizations of the predicted *versus* actual values were generated for the datasets. These visualizations enabled a clear comparison of how well the model fitted the data, particularly in terms of overfitting (if present in the training set) or underfitting (in the validating set). Additionally, learning curves were plotted to observe the models' convergence behaviors over different training set sizes, offering insights into their performances with increasing data. The learning curves were constructed using a series of training set sizes, ranging from 10 to 100% of the data. Lastly, we tested the performance of the model with all test data instead of part of the test data in K-fold. It is useful for evaluating the final performance of an ML model on unseen (test) data and for assessing how well the model generalizes beyond the training and validation phases.

### 2.6. Synthesis and characterization of AuNS

To grow AuNS with short branches and one LSP band, HAuCl_4_ (final concentration of 0.2 mM) was mixed with a HEPES solution (pH 7.4, final concentration of 150 mM, and final volume of 10 mL), vigorously stirred, and left undisturbed for 2 h. HEPES was purchased from Carl Roth (Karlsruhe, Germany), and HAuCl_4_·3H_2_O was purchased from Sigma-Aldrich (St. Louis, USA). The AuNS extinction spectra were measured with a TECAN Infinite M200 Pro microplate reader (Tecan Group Ltd, Männedorf, Switzerland), and their morphology was characterized with a Hitachi transmission electron microscope at 100 kV.

## 3. Results and discussion

### 3.1. Feature initial evaluation and selection

**Fig. 3:**
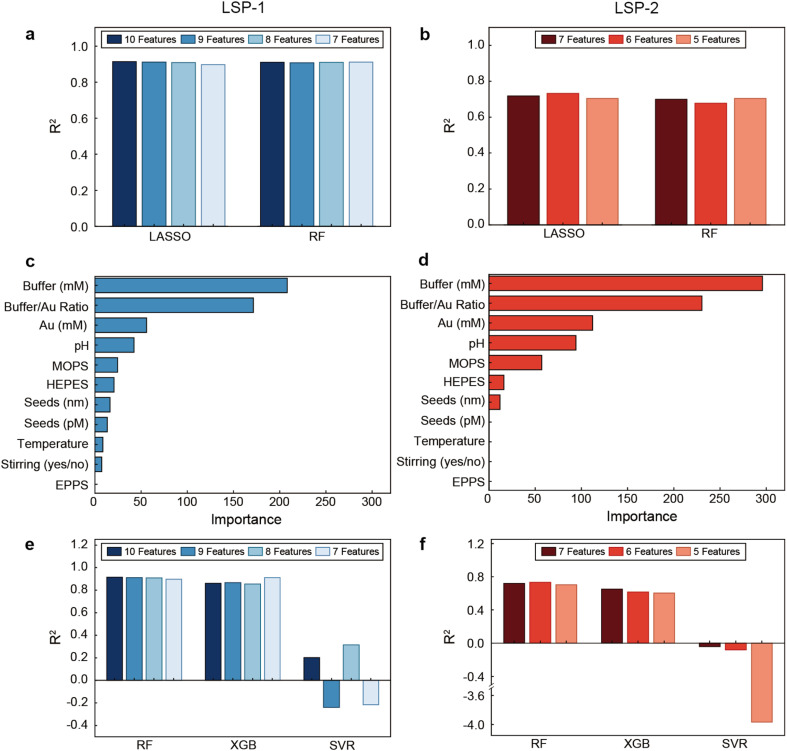
Selection of features. (a) *R*^2^ calculated with the best model, RF, using different numbers of features selected with LASSO or RF for LSP-1 prediction. (b) *R*^2^ calculated with the best model, RF, using different numbers of features selected with LASSO or RF for LSP-2 prediction. Importance ranking of all features for (c) LSP-1 and (d) LSP-2 prediction. (e) *R*^2^ calculated with all models using different numbers of features selected with LASSO for LSP-1 prediction. (f) *R*^2^ calculated with all models using different numbers of features selected with LASSO for LSP-2 prediction.

To identify the most effective feature selection algorithm, we compared RF and LASSO as feature selectors across the three models (RF, XGB, and SVR) and with bootstrapping as data generator, as it had been successfully used in the analysis of gold nanoparticle synthetic parameters.^38^ The best feature selection algorithm should achieve the highest *R*^2^ or lowest error while minimizing feature numbers, and should align with empirical observations. Our results indicate that all models achieved their highest performance with LASSO (Fig. 3a and S3†).

For example, for the prediction of LSP-1 with the RF model (the one with the best performance), although both selector methods showed relatively high *R*^2^ values (around 0.9) with different feature numbers (Fig. 3a), the RF model with ten features selected by LASSO was the combination with the best performance (*R*^2^ value of 0.914). Similarly, for LSP-2 prediction, the combination of LASSO with six features was the one with the highest *R*^2^ value (0.733) (Fig. 3b). Taken together, these results pointed out that the RF model with feature selection based on LASSO performed the best when predicting both LSP-1 and LSP-2, even though the prediction of the first plasmonic band was better, as its *R*^2^ value was higher (0.914 and 0.733 for LSP-1 and LSP-2, respectively). This was likely caused by the different data sizes for both plasmonic bands, since all AuNS display LSP-1, but only a small fraction of particles possesses LSP-2 (original data size of 144 and 32, respectively). Hence, based on the comparative analysis, LASSO emerged as the superior feature selection method, which led us to adopt it for the subsequent analyses.

Identifying the most relevant features and evaluating their importance is essential to optimize the predictive models. Fig. 3c and d present the feature importance rankings determined using LASSO for LSP-1 and LSP-2, respectively. These rankings, which include both numerical and categorical features, illustrate the contribution of each feature to the prediction tasks. The importance rankings identified ten features for LSP-1 (*i.e.* buffer concentration (mM), buffer/gold concentration ratio, gold concentration (mM), pH, buffer type MOPS, buffer type HEPES, seed size (nm), seed concentration (pM), temperature (°C), and stirring (yes/no)) and seven features for LSP-2 (*i.e.* buffer type MOPS, buffer type HEPES, buffer/gold concentration ratio, pH, buffer concentration (mM), gold concentration (mM), and seed concentration (pM)) as the most relevant to predict the plasmon band positions, which are consistent with qualitative experimental observations previously reported.^20–22^

Based on these features, we trained the three regression models, namely RF, XGB, and SVR, and the selection method LASSO, to identify the best prediction model and the appropriate number of features. Consistent with our previous results, the RF model achieved the highest *R*^2^ values with ten (up to *R*^2^ of 0.914) and six (up to *R*^2^ of 0.733) features for predicting LSP-1 and LSP-2, respectively (Fig. 3e and f), while the SVR model severely underperformed, with some instances showing negative *R*^2^ values, indicating very poor predictions.

Understanding the relationships between features is essential to validate and refine the feature selection. We initially assessed potential redundancies or strong interactions of features based on correlation matrices and pair plot diagrams and identified the relationships between features and their potential predictive power (Fig. S4 and S5†). For example, seeds (nm) were negatively correlated with LSP-1 (nm) with a Pearson correlation coefficient (*r*) of −0.35, and buffer (mM) was positively correlated with LSP-1 (nm) and LSP-2 (nm) with *r* of 0.51 and 0.48, respectively, suggesting that they are important features for prediction. Moreover, the feature buffer concentration (mM) and the buffer/gold concentration ratio were almost perfectly correlated (*r* of 0.96). The features seed (nm) and seed (pM), which refer to the physical size and concentration of the seed, respectively, were also highly correlated (*r* of 0.86), indicating that they convey very similar information. In ML, using two highly correlated features simultaneously may lead to multicollinearity problems. Therefore, we compared the prediction results of excluding the seed (nm) feature and buffer/gold concentration ratio. The results showed that retaining these two highly correlated features led to better model performance (Table S1†).

In conclusion, our analysis demonstrates that LASSO is the optimal feature selection method for our study, with LSP-1 requiring ten features and LSP-2 six features for achieving the best prediction outcomes.

### 3.2. Data generation

**Fig. 4:**
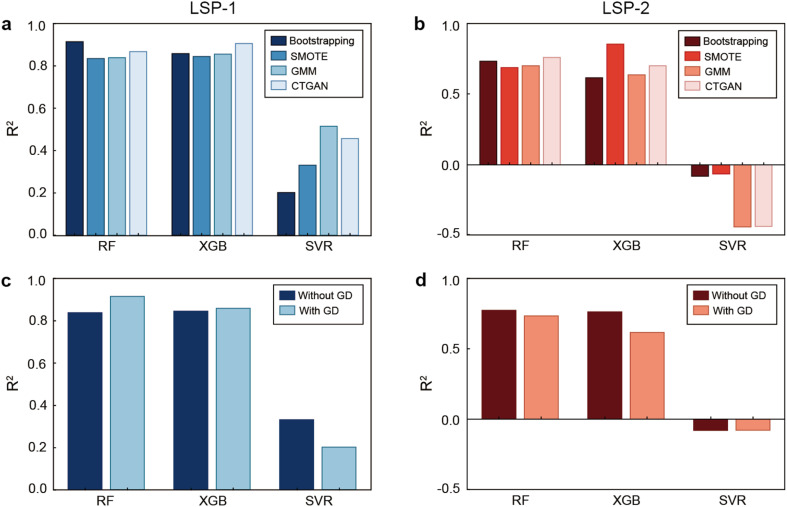
Impact of data generation. Performance of all models using different methods of data generation for (a) LSP-1 and (b) LSP-2. *R*^2^ metrics for models without and with generated data (with bootstrapping) for (c) LSP-1 and (d) LSP-2. The values displayed refer to test dataset predictions. For performance breakdown based on training and validating sets refer to Tables S2 and S3.† GD stands for generated data.

To further improve the performance of the ML algorithms in predicting the LSP band positions, we optimized the data augmentation strategy. For this purpose, we combined the three regression algorithms (RF, XGB, and SVR) with four data generation methods, namely bootstrapping, SMOTE, GMM, and CTGAN. For LSP-1, all four data generation methods performed well when combined with the RF and XGB models, with *R*^2^ values above 0.830 (Fig. 4a). Among them, we confirmed that the bootstrapping method combined with the RF model gave the best result with an *R*^2^ value of 0.914. For LSP-2, three combinations displayed better results, namely the XGB model combined with SMOTE, the RF model combined with CTGAN, and the RF model combined with bootstrapping with *R*^2^ values of 0.855, 0.759, and 0.733, respectively (Fig. 4b).

To further understand the impact of generated data on the models' performance, we compared their performance based on three metrics, namely *R*^2^ value, MAE, and MSE. Furthermore, we broke down the performance of the algorithms into seen data (training set in each cross-validation fold without or with synthetic data), unseen data (validating set in each cross-validation fold), and full unseen data (test dataset). While the performance of all algorithms improved during the prediction of the training and testing LSP-1 data after data augmentation, the most significant changes occurred when data generation was applied to predict the validating datasets (Table S2†). For instance, the *R*^2^ value for the validating data using the three models only increased with bootstrapping synthetic data (from 0.716 to 0.955, from 0.533 to 0.962, and from 0.378 to 0.480 for the RF, XGB, and SVR models, respectively). The impact of the generated data on predicting the validating sets was also observed in the MAE and MSE values, as they all significantly decreased after implementing the bootstrapping data generation (Table S2†). Similar results were observed for the LSP-2 position prediction, as the performance of all algorithms significantly improved after data augmentation in all training and most validating data sets (Table S3†). Regarding the prediction of the test datasets, the *R*^2^ metrics for all three models trained with original data and those trained with both original and generated (with bootstrapping) data for LSP-1 and LSP-2 were compared (Fig. 4c and d). The RF model provided the best predictions for LSP-1 and LSP-2. For LSP-1, the *R*^2^ value increased from 0.837 to 0.914 after data generation. For LSP-2, the *R*^2^ value decreased from 0.773 to 0.733. However, the metric MAE, decreased after the data generation, proving the contribution of synthetic data in improving the prediction results (test set). Hence, the combination of the RF model and the bootstrapping method was considered the best option moving forward, since it performed the best for LSP-1 prediction and was one of the best when predicting LSP-2.

Next, we analyzed the similarity between the original data and the generated data by the bootstrapping method. To assess the similarity between the original and bootstrapped datasets, we performed the Kolmogorov–Smirnov (KS) test for each feature. The KS statistic quantifies the largest difference between the cumulative distributions of the two datasets, while the *p*-value assesses the statistical significance of this difference. Fig. S6 and S7† compare the distributions of the most important features between both datasets for LSP-1 and LSP-2, confirming that the generated data by bootstrapping strongly resembled the original one with *p*-values of 1.00 and KS values below 0.03.

Overall, the results illustrate that RF consistently provided the most reliable predictions for both LSP-1 and LSP-2, while XGB also performed well but tended to underperform on the test and validating datasets (Tables S2†). SVR demonstrated poor performance due to the non-linear relationship between the features. Moreover, all regression algorithms were better at predicting the position of the first plasmon band than the second one. As previously discussed, this may be caused by the greater number of data points for LSP-1, since all AuNS display the first plasmonic band, but many do not present a second one.

To better characterize the impact of data augmentation on the best-performing model, particularly on convergence and generalization, we compared the learning curves of the RF model trained without and with generated data using bootstrapping. We selected MAE for plotting the learning curves due to its interpretability, robustness to outliers, and ability to directly assess the average prediction error. These graphs depict two important aspects, namely training error and cross-validation error. The former represents how well the model performs on the data it has been exposed to, while the latter estimates the model's ability to generalize to unexposed data. The gap between the two curves indicates the degree of overfitting or underfitting, while the shaded area around the training and test MAE curves represents variability or spread of error, usually due to the inherent variability of the model predictions across subsets of data, visually assessing the stability of the model performance.

**Fig. 5:**
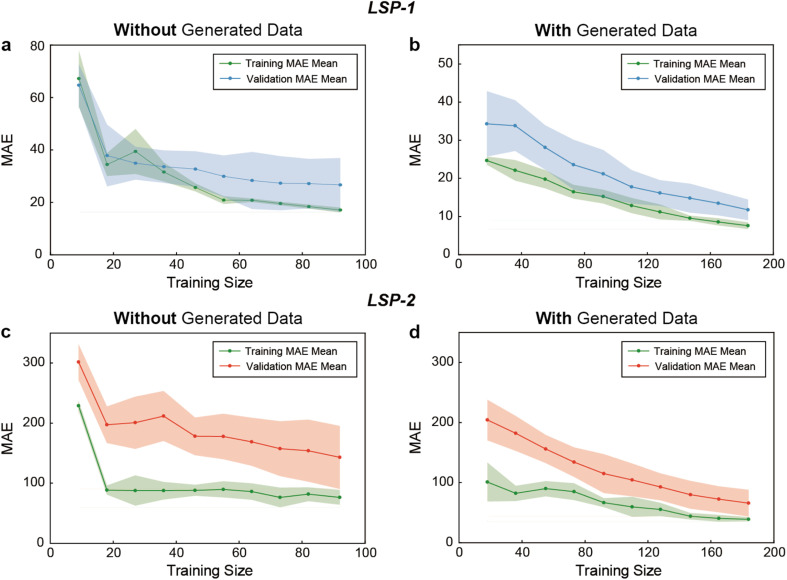
Evaluation of convergence and generalization. Learning curves of LSP-1 prediction by the RF model (a) without and (b) with data generation. Learning curves of LSP-2 prediction by the RF model (c) without and (d) with data generation. The shaded regions around the training and cross-validation. Data generation was performed with bootstrapping. MAE curves correspond to standard deviations above and below the mean of MAE.

Fig. 5a shows the learning curves of LSP-1 without data generation. As the number of training examples increased to 92, the training MAE decreased to 17.0. The validation MAE followed a similar trend, starting at 64.6 and stabilizing around 26.6 for larger datasets, suggesting some overfitting. In Fig. 5b, the learning curves for the RF model predicting LSP-1 showed faster convergence and reduced error rates when data generation was applied in the validating set. Initially, the training MAE was 24.7, but then dropped rapidly to 7.6, and the validation MAE stabilized at 11.8, which was significantly lower than it was without data generation. The shaded area also narrowed, indicating less variability in model performance, most likely due to the data addition of the training set. Similar learning speed and generalization improvements were observed for the RF model predicting LSP-2, further demonstrating the positive impact of data generation. In Fig. 5c, the model showed higher error values for LSP-2 without generating data. The training MAE started at 228.8 and decreased to 76.6 as the number of training examples increased to 80. The validation MAE showed a similar trend, but ended up with a higher error, 143.0, which suggested that the model suffered from overfitting and struggled to generalize to unseen data. Fig. 5d shows the impact of data generation on LSP-2 prediction. The training MAE started at 100.9 and steadily decreased to 39.1 as the data increased. The validation MAE decreased accordingly and stabilized at 65.9 for larger dataset sizes. Compared with Fig. 5c, the shaded area in Fig. 5d was narrower, indicating a more stable model performance with less variation in MAE, reflecting better generalization.

**Fig. 6:**
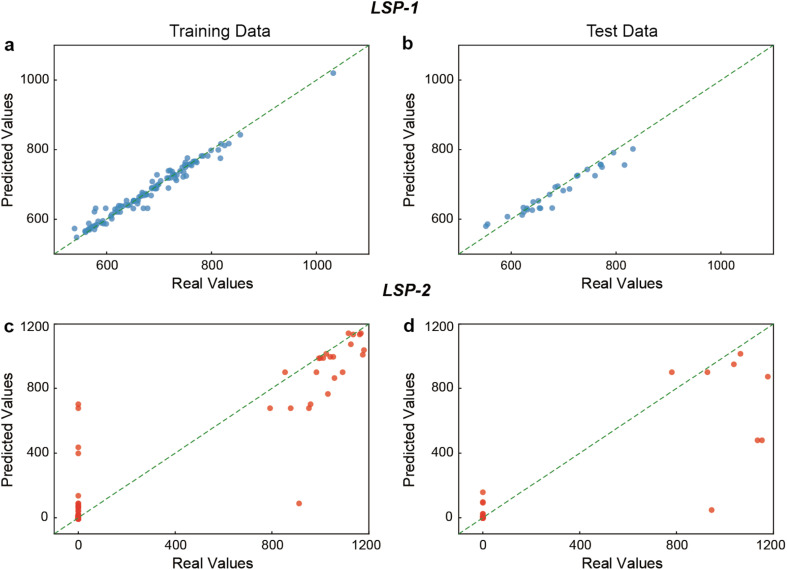
Assessing the predictive performance of the RF model with scatter plots. Visualizing the prediction of the LSP-1 position by the RF model with bootstrapping data generation in the (a) training and (b) test sections. Visualizing the prediction of the LSP-2 position by the RF model with bootstrapping data generation in the (c) training and (d) test sections.

Next, because the RF model performed the best when combined with LASSO feature selection and bootstrapping data augmentation, we assessed its generalization capacity with scatter plots. For the training data, the scatter plot shows a very good alignment along the diagonal (Fig. 6a), resulting in a high degree of accuracy in predicting LSP-1 values within the training set (*R*^2^ and MSE of 0.980 and 145.7, respectively, Table S2†). For the test set, the data points were more dispersed than in the training set but still well aligned with the diagonal (Fig. 6b), reflecting good generalization of the model to unseen data for the LSP-1 (*R*^2^ and MSE of 0.914 and 455.4, respectively, Table S2†). For the LSP-2, the scatter plots show more variance around the diagonal in both training and test sets compared to LSP-1, especially at higher values (Fig. 6c and d), indicating that the model has more difficulty predicting LSP-2 in both datasets (Table S3†). These observations further confirmed our previous observation that the RF model is less effective when predicting the second plasmon band position.

### 3.3. Multi-output model

Up to this point, the positions of LSP-1 and LSP-2 had been independently predicted (one at a time). Nevertheless, it would be highly beneficial if the model could be used to predict both plasmon bands simultaneously. Hence, in this last section, we explored whether the RF model combined with LASSO feature selection and bootstrapping data generation could be further used to predict both LSP-1 and LSP-2 simultaneously.

**Fig. 7:**
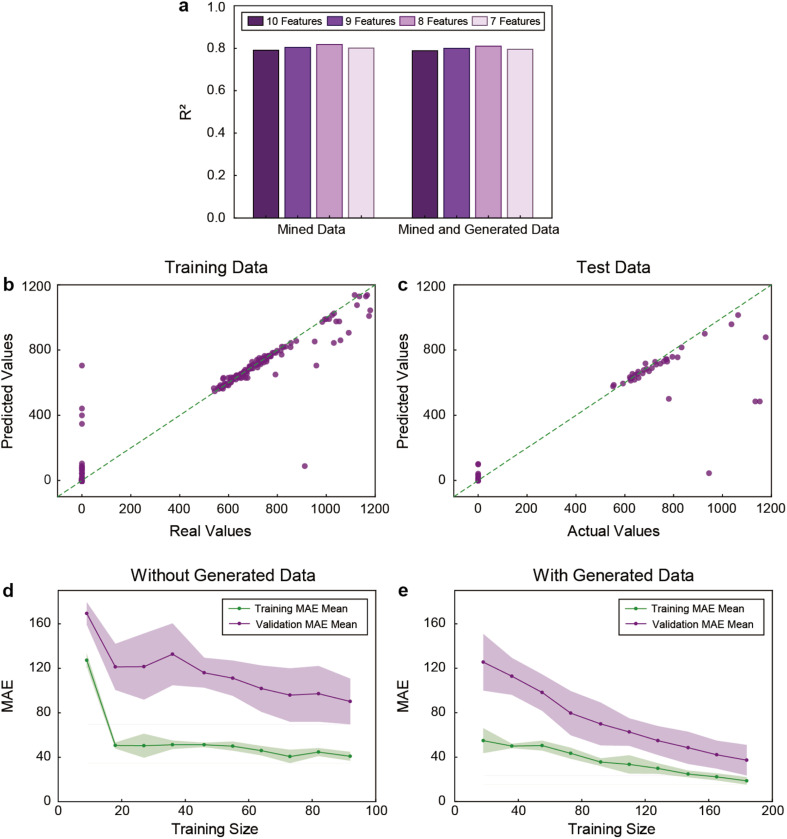
Evaluation of the multi-output model performance. (a) *R*^2^ calculated for test dataset with multi-output RF with and without bootstrapping generated data using different numbers of features selected with LASSO. Visualizing the prediction of the multi-output RF model with data generation with bootstrapping in the (b) training and (c) test sections. Learning curves of the multi-output RF model (d) without and (e) with data generation, respectively.

First, we assessed the optimal number of features necessary for LSP-1 and LSP-2 prediction with the multi-output RF model. Fig. 7a compares the model performance when trained with the original data alone and when combined with the generated data (with bootstrapping), using feature sets ranging from 7 to 10 features. The results obtained with eight features were the best for both predictions. These features were buffer type MOPS, buffer type HEPES, pH, buffer concentration (mM), ratio of buffer/gold concentration, gold concentration (mM), seed concentration (pM), and temperature (°C) (in descending order of importance), and were consistent with the ones previously identified for single LSP bands. Notably, the model trained with the combined original and generated data demonstrated better performance, indicating that the inclusion of the synthetic data improved the model training process.

To further analyze the performance of multi-output RF after data generation, we calculated its metrics with eight features using the original and generated data (Table S4†). When trained using only original data, the RF model achieved an *R*^2^ value of 0.894 on the training set, 0.638 on the validating set, and 0.817 on the test dataset. Nevertheless, the MAE and MSE values indicated relatively high errors in both the training and test phases. In contrast, the RF model improved in most metrics when trained with both original and generated data. The *R*^2^ value increased to 0.965 and 0.902 in the training and validating sets, respectively, though its performance in test datasets slightly decreased (0.809). The MAE and MSE values were also reduced in training sets, where the MAE and MSE decreased by 56.2% and 56.1%, respectively, a significant improvement compared to using original data alone. Therefore, these results showed that the inclusion of generated data improved the training performance of the model and overall reduced errors. However, the *R*^2^ improvement across the validating and test datasets was not significant. This suggests that while data generation is beneficial, in this case the benefits are reduced when applied to entirely unseen data.

After confirming that the feature selection and data generation methods are applicable (and potentially beneficial) to the multi-output RF, we visualized the prediction and generalization of the multi-output model in the training and test sections with scatter plots. For training data, a strong alignment between the predicted and actual values was observed (Fig. 7b). Notably, most data points clustered tightly around the diagonal red line, representing good predictions and suggesting that the model performed well on the training set (*R*^2^ value of 0.965 and an MSE value of 4327.6, Table S4†). For test data, while the alignment with the diagonal red line was less pronounced, it remained relatively strong (Fig. 7c). The *R*^2^ value of 0.809, and the MSE value of 33 616.1 suggested that the model performance on the test data was acceptable (Table S4†).

Lastly, we analyzed the model performance regarding MAE through its learning curves. For only original data (without synthetic data), as the number of training examples increased, the training MAE quickly stabilized around 40, indicating that the model fitted the training data well (Fig. 7d). Nevertheless, the validation MAE remained significantly higher, hovering around 100 to 140, and fluctuating significantly as more examples were used. The gap between the training MAE and the validation MAE suggested potential overfitting, where the model performed well on the training set but underperformed when generalized to unseen data. When the original data was enriched with generated data (with bootstrapping), the curves showed similar patterns, but improved learning due to the inclusion of the synthetic data (Fig. 7e). The faster convergence and smaller gap between the training MAE and the validation MAE curves suggested that the model benefited from the data generation, which enhanced its ability to generalize to unseen data, further validating the approach. In summary, the multi-output RF model with data generation demonstrated a strong predictive performance on both training and validating datasets. However, the performance on the test set exhibited a greater variability, reflecting the typical decrease in model accuracy when generalizing to unseen data.

### 3.4. Discussion

This study provides important insights into feature selection and data generation methods for predicting the LSP position of AuNS using ML models. LASSO-based feature selection and RF feature importance rankings helped to identify key predictors for LSP-1 and LSP-2, which included buffer concentration (mM), buffer/gold concentration ratio, gold concentration (mM), pH, buffer type MOPS, buffer type HEPES, seed size (nm), seed concentration (pM), temperature (°C), and stirring (yes/no). Notably, the EPPS buffer showed no importance for predicting both LSP-1 and LSP-2 (Fig. 3a and b). This was likely due to the chemical similarities between EPPS and HEPES, which differ only by a single carbon in the sulfonic acid chain. In contrast, MOPS has a distinct chemical structure, lacking the hydroxyethyl chain. These findings align with the experimental results, which indicate that EPPS and HEPES yield very similar AuNS, while MOPS produces more anisotropic and distinct nanocrystals.^19,20^

The decision to apply data generation techniques, such as bootstrapping, played a crucial role in improving the model performance. Our results indicated that synthetic data generation generally improved the models' accuracy. This was particularly evident when comparing models trained with original data or with both original and generated data. For instance, the RF model performance for LSP-1 improved significantly with data generation, as reflected by the higher *R*^2^ values and lower MAE and MSE metrics. However, for LSP-2, while data generation also improved performance, the gains were less pronounced, particularly on the test dataset, as the *R*^2^ values were lower. This may be explained by particles infrequently displaying LSP-2, since all AuNS possess LSP-1, but only a small fraction of those display LSP-2. Thus, the original dataset for LSP-2 was smaller, which could lead to data aggregation and incomplete information, resulting in less effective predictions. To enhance model training and improve overall predictive performance, future efforts should focus on generating larger datasets that include AuNS exhibiting a second plasmon band. This expanded data will allow the models to capture better the complex behaviors associated with multi-plasmonic responses.

Regarding model selection, RF consistently outperformed the other models, namely XGB and SVR, especially for LSP-1 prediction. The RF model was suitable for this study because of its ability to handle non-linear interactions and robustness against overfitting. However, for LSP-2, the performance of all models, including RF, was slightly lower, suggesting that more exploration of feature correlations or more sophisticated data generation techniques may be required.

Lastly, the multi-output RF model demonstrated strong predictive performance on both training and validating datasets after data generation. Greater variability was identified when predicting the test data, which was consistent with lower model accuracy when generalizing to unseen data.

## 4. Conclusions

In summary, we have applied ML models, namely RF, XGB, and SVR, to predict the LSP-1 and LSP-2 positions of AuNS based on features that had been reported to influence them during synthesis. Feature importance evaluation and selection were carried out with the LASSO and RF methods, where the former performed better, identifying buffer concentration (mM), buffer/gold concentration ratio, gold concentration (mM), pH, buffer type MOPS, buffer type HEPES, seed size (nm), seed concentration (pM), temperature (°C), and stirring as the most critical parameters affecting the AuNS optical properties. Because the models were trained with a relatively small data set (144 samples), synthetic data was generated using four different methods (bootstrapping, SMOTE, GMM, and CTGAN). The inclusion of synthetic data during training consistently improved the prediction results for all models, with specific combinations performing better for the prediction of LSP-1 (RF and bootstrapping with *R*^2^ of 0.914) and LSP-2 (XGB and SMOTE with *R*^2^ of 0.855). The ML workflow made possible simultaneous predictions of both LSP band positions, but the prediction performance decreased (*R*^2^ of 0.817). In summary, these results illustrate the use of ML models to predict the optical properties of AuNS synthesized through seedless synthesis, which may offer new opportunities for improving the nanoparticle design and their final applications.

## Data availability

The data supporting this article have been included as part of the ESI.†

## Conflicts of interest

All authors declare that they have no conflicts of interest.

## Supplementary Material

NA-OLF-D5NA00265F-s001
