# Supplementary material for: Machine learning to predict gold nanostar optical properties
Source: Nanoscale Adv. 2025 May 27;7(13):4117–28. doi: 10.1039/d5na00265f (PMC12108964; doi:10.1039/d5na00265f)
Supplement: NA-007-D5NA00265F-s001 [file NA-007-D5NA00265F-s001.pdf]

Supplementary Information (SI) for Nanoscale Advances.
This journal is © The Royal Society of Chemistry 2025

# - Supplementary Information -

Machine Learning to Predict Gold Nanostar Optical Properties

Peiying Wu,[1] Rui Zhang,[1] Celine Porte,[1] Fabian Kiessling,[1,2] Twan Lammers,[1] Sima Rezvantalab,[3,*] Sara Mihandoost,[4,*] and Roger M. Pallares[1,*]

[1]Institute for Experimental Molecular Imaging, RWTH Aachen University Hospital, Aachen 52074, Germany

[2]Fraunhofer Institute for Digital Medicine MEVIS, Bremen 28359, Germany

[3]Chemical Engineering Department, Urmia University of Technology, Urmia 57166-419, Iran

[4]Electrical Engineering Department, Urmia University of Technology, Urmia 57166-419, Iran

[*]Corresponding author: rmoltopallar@ukaachen.de, s.rezvantalab@uut.ac.ir, s.mihandoost@uut.ac.ir

Table of contents

Figure S1. Molecular structure of Good's buffers. 2

Figure S2. The distribution plot of the target variables. 3

Figure S3. Feature selection algorithm evaluation. 4

Figure S4. The Pearson correlation matrix. 5

Table S1. Comparison of excluding features for LSP-1 prediction with RF model. 7

Figure S6. Distribution of all numeric features in original dataset and generated data for LSP-1. 9

Figure S7. Distribution of all numeric features in original dataset and generated data for LSP-2. 10

Table S2. Evaluation metrics for the training and test set using original and generated data for LSP-1. 11

Table S3. Evaluation metrics for the training and test set using original and generated data for LSP-2. 12

Table S4. Evaluation metrics of the multi-output RF model using original and generated data. 13

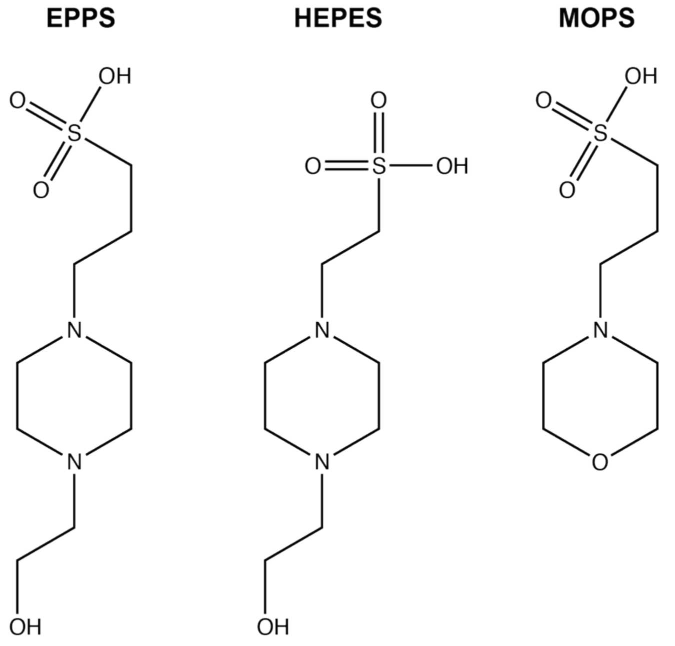

Figure S1. Molecular structure of the Good's buffers.

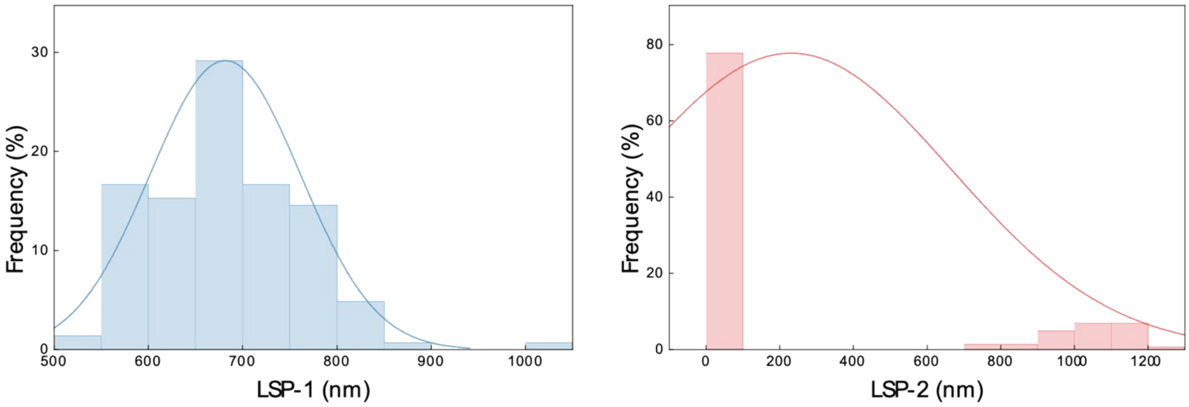

Figure S2. The distribution plot of the target variables.

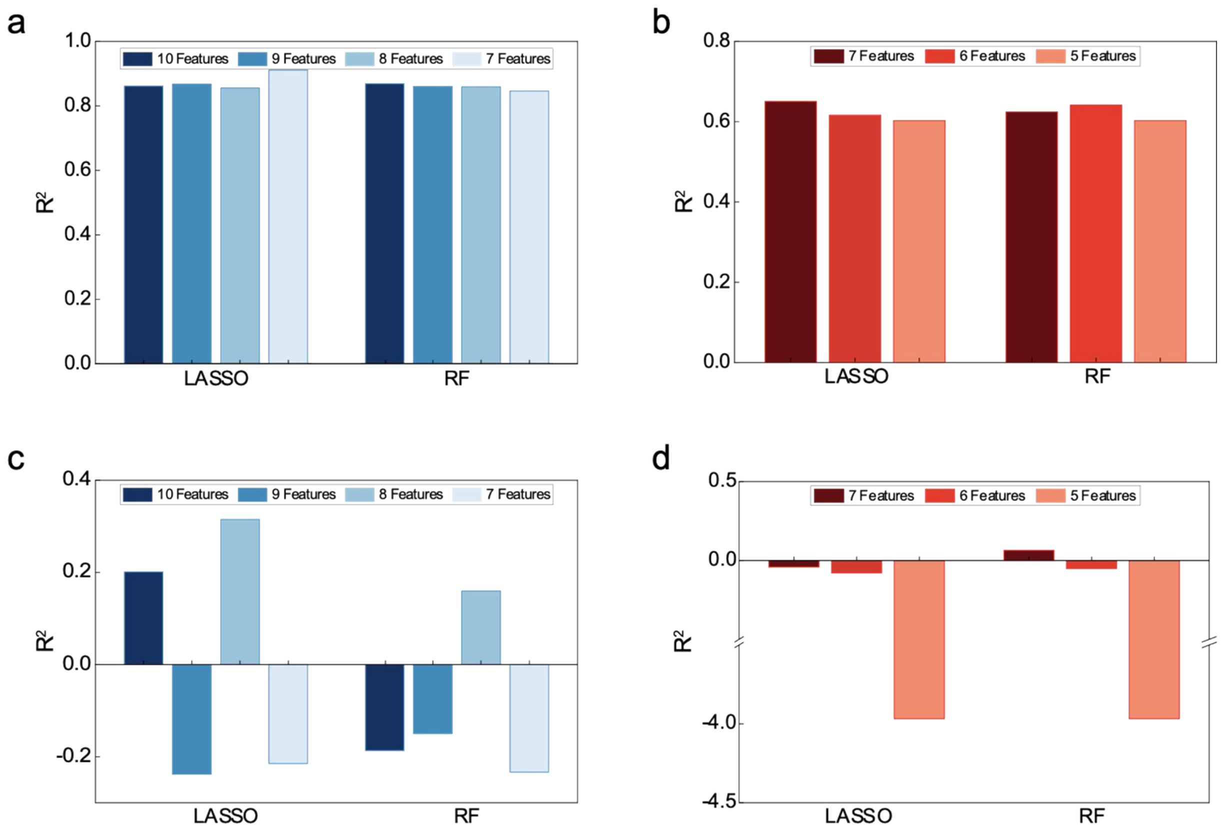

Figure S3. Feature selection algorithm evaluation. R^2 calculated with the XGB model, using different numbers of features selected with LASSO or RF for (**a**) LSP-1 and (**b**) LSP-2 prediction. R^2 calculated with the SVR, using different numbers of features selected with LASSO or RF for (**c**) LSP-1 and (**d**) LSP-2 prediction.

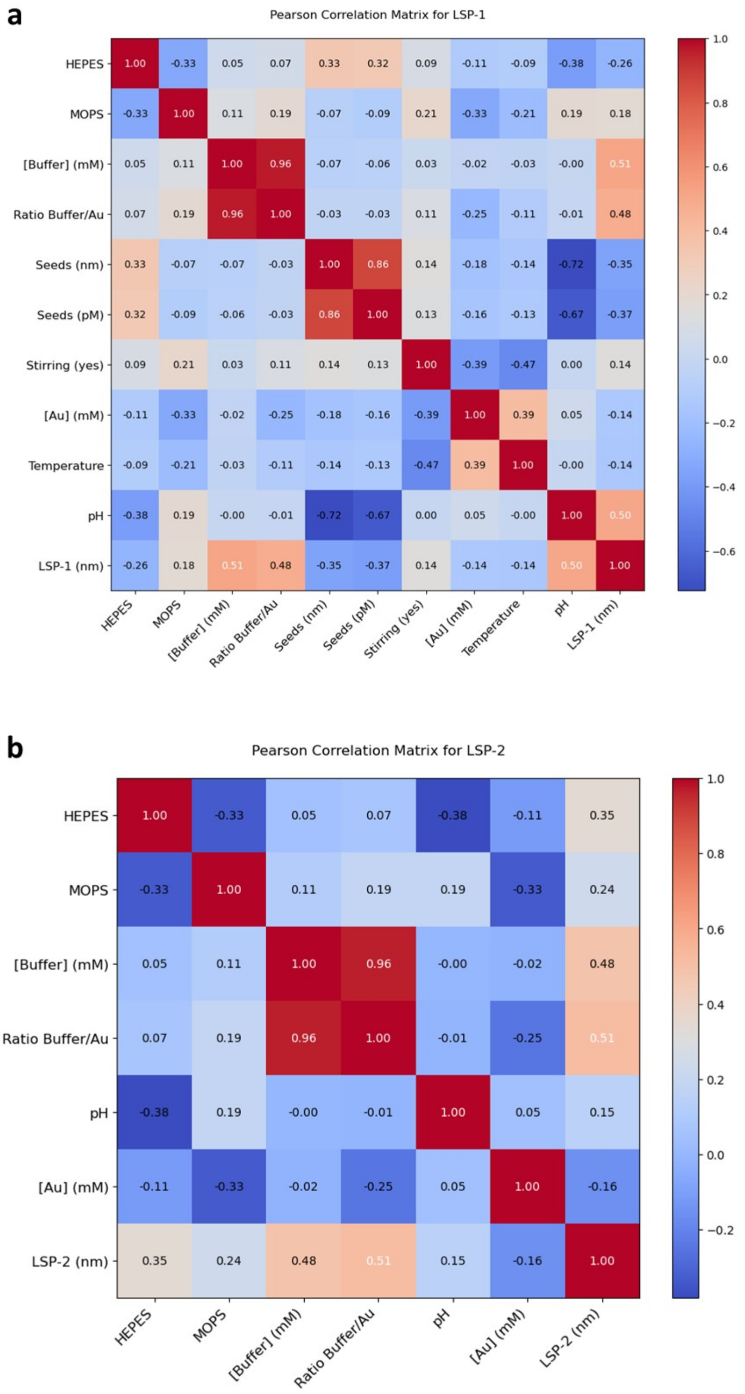

Figure S4. The Pearson correlation matrix. (**a**) Matrix for LSP-1 with 10 features, and (**b**) matrix for LSP-2 with 6 features.

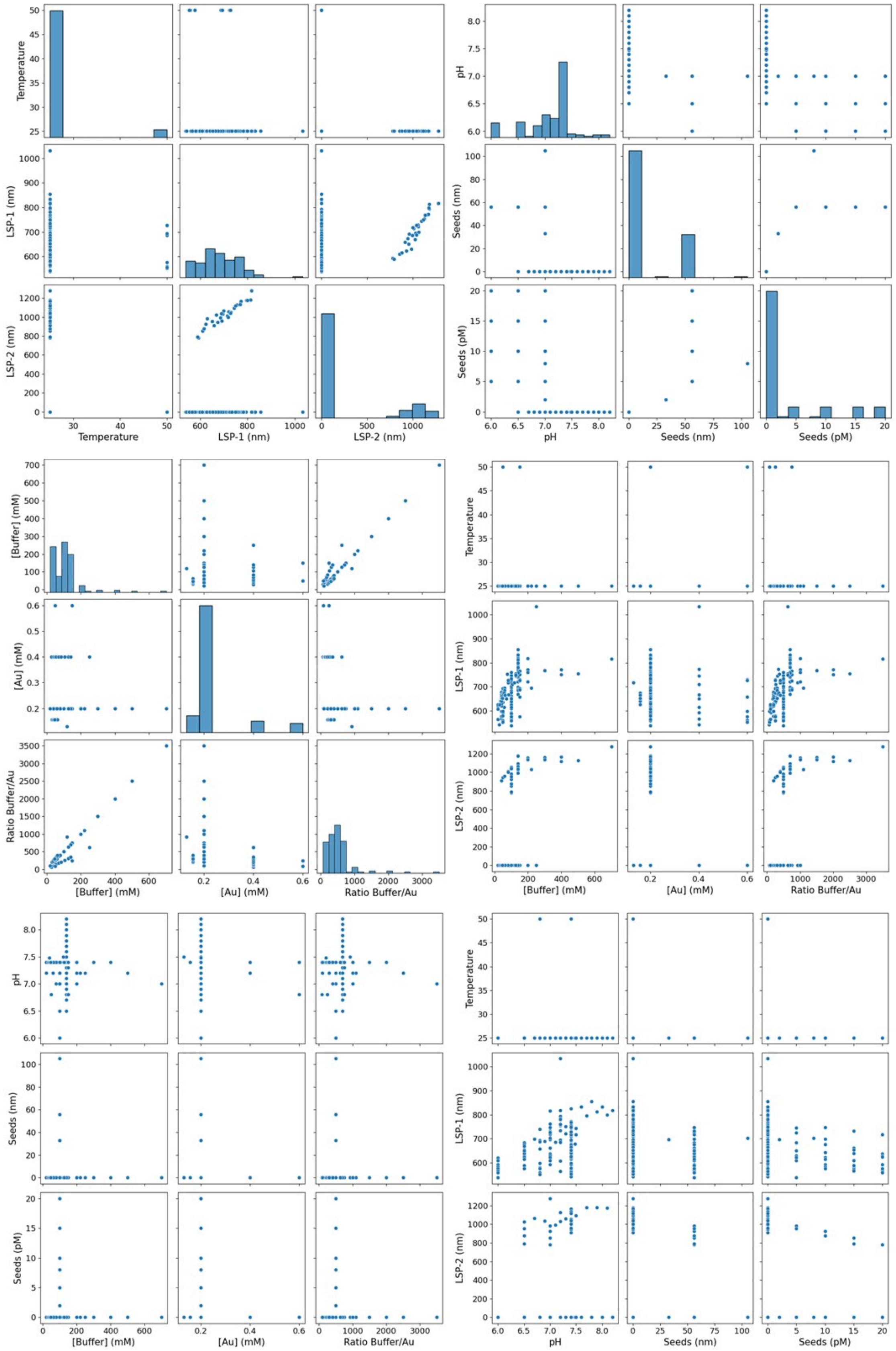

Figure S5. Pair plot for the 9 numerical descriptors. The bar charts show the distribution of one feature and the scatter plots show the relationship between two features.

Table S1. Comparison of excluding the features ratio buffer/Au and seed (nm) for LSP-1 prediction with RF model.

Model	Training set (mean)			Validating set (mean)			Test set (mean)		
	R^2	MAE	MSE	R^2	MAE	MSE	R^2	MAE	MSE
10 features	0.980	7.2	145.7	0.955	11.3	303.4	0.914	15.7	455.4
Excluding buffer/Au ratio	0.976	8.0	175.0	0.939	12.7	409.7	0.890	18.9	585.8
Excluding seed (nm)	0.968	9.0	231.3	0.945	12.3	375.1	0.898	17.5	544.0

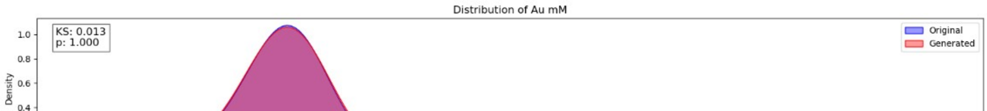
Distribution of Au mM
KS: 0.013
p: 1.000
Original
Generated
Density
1.0
0.8
0.6
0.4

Figure S6. Distribution of all numeric features in original dataset and generated data for LSP-1. Buffer type 1 and 2 refer to HEPES and MOPS, respectively. The KS statistic quantifies the largest difference between the cumulative distributions of the two datasets, while the p-value assesses the statistical significance of this difference.

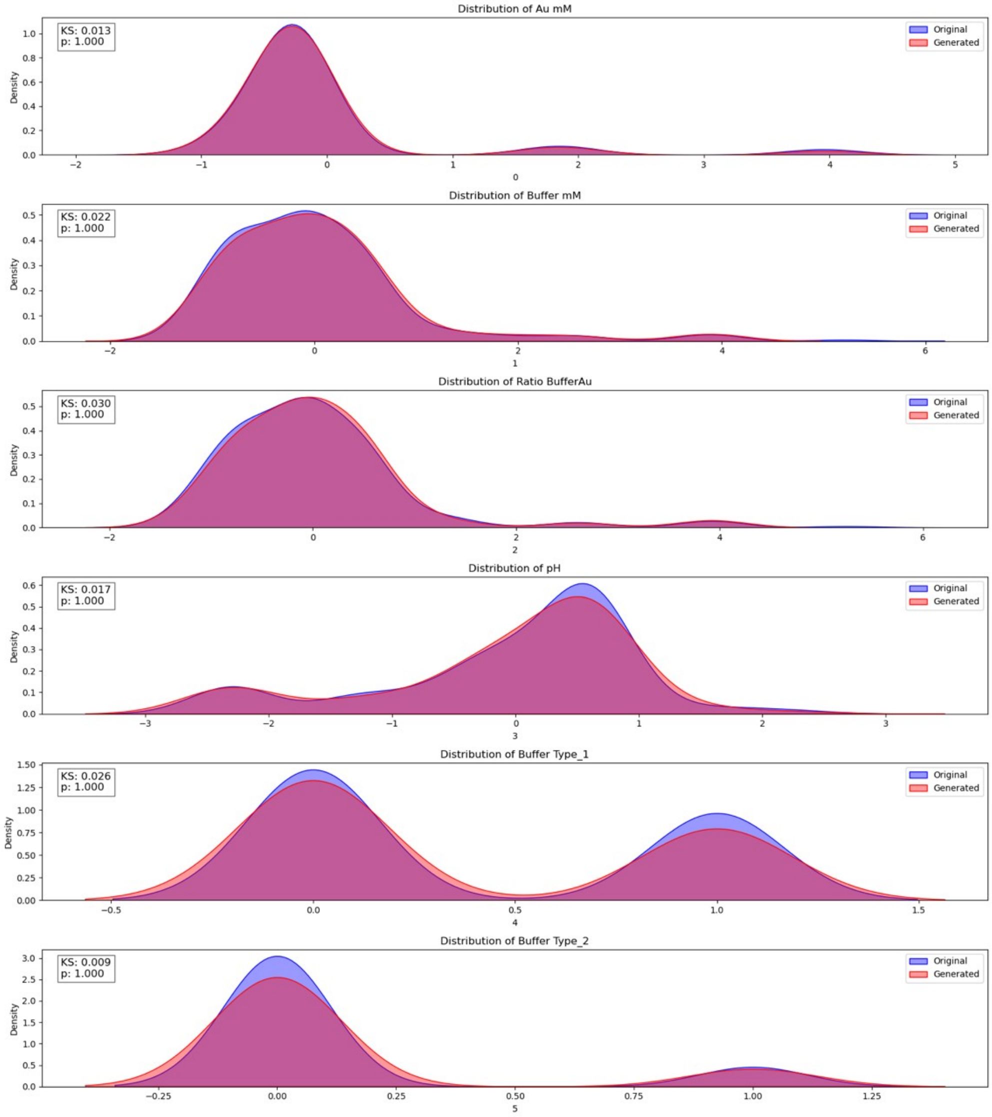

Figure S7. Distribution of all numeric features in original dataset and generated data for LSP-2. Buffer type 1 and 2 refer to HEPES and MOPS, respectively. The KS statistic quantifies the largest difference between the cumulative distributions of the two datasets, while the p-value assesses the statistical significance of this difference.

Table S2. Evaluation metrics for the training and test set using original and generated data for LSP-1.

Model	Database		Training set (mean)			Validating set (mean)			Test set (mean)		
	Metrics		R^2	MAE	MSE	R^2	MAE	MSE	R^2	MAE	MSE
RF	Original data		0.923	16.6	504.5	0.716	26.6	1879.8	0.837	22.7	864.6
	Original and generated data	bootstrapping	0.980	7.2	145.7	0.955	11.3	303.4	0.914	15.7	455.4
		SMOTE	0.922	16.2	496.1	0.703	26.1	1826.1	0.835	22.7	878.0
		GMM	0.932	13.1	491.9	0.206	50.5	5581.4	0.839	22.1	858.5
		CTGAN	0.781	28.8	1577.1	0.234	51.7	5431.9	0.868	20.5	703.5
XGB	Original data		0.980	7.3	133.6	0.533	27.2	2807.9	0.845	20.6	826.7
	Original and generated data	bootstrapping	0.990	2.5	69.3	0.962	7.0	245.5	0.858	20.7	756.8
		SMOTE	0.980	7.3	133.6	0.533	27.2	2807.9	0.845	20.6	826.7
		GMM	0.992	3.7	55.9	0.219	50.7	5524.3	0.856	19.0	767.9
		CTGAN	0.994	2.3	40.7	0.142	52.1	6073.3	0.906	16.4	501.6
SVR	Original data		0.546	36.4	2979.3	0.378	41.1	3527.7	0.331	38.1	3557.5
	Original and generated data	bootstrapping	0.936	12.5	461.7	0.480	20.7	3093.4	0.201	33.5	4247.4
		SMOTE	0.546	36.4	2979.3	0.378	41.1	3527.7	0.331	38.1	3557.5
		GMM	0.160	54.8	6056.3	0.108	57.5	6408.7	0.515	38.6	2580.8
		CTGAN	0.143	57.9	6180.7	0.026	61.5	6762.0	0.457	41.8	2889.5

Table S3. Evaluation metrics for the training and test set using original and generated data for LSP-2.

Model	Database		Training set (mean)			Validating set (mean)			Test set (mean)		
	Metrics		R^2	MAE	MSE	R^2	MAE	MSE	R^2	MAE	MSE
RF	Original data		0.858	80.0	23925.5	0.522	148.1	74809.4	0.773	131.6	55417.5
	Original and generated data	bootstrapping	0.921	40.5	13321.3	0.825	58.2	28094.5	0.733	119.3	65390.5
		SMOTE	0.866	78.7	23623.2	0.545	126.7	59295.1	0.688	145.9	76202.7
		GMM	0.864	86.4	22865.0	0.075	232.0	147067.2	0.701	137.1	73199.4
		CTGAN	0.891	78.2	18345.5	0.150	228.6	132603.9	0.759	121.3	58848.0
XGB	Original data		0.954	19.9	7800.5	0.555	128.8	73978.6	0.762	117.1	58192.8
	Original and generated data	bootstrapping	0.936	26.6	10783.4	0.828	53.0	27526.3	0.616	128.0	93852.9
		SMOTE	0.923	38.7	13129.0	0.507	137.9	85588.1	0.855	80.1	35403.7
		GMM	0.962	17.5	6442.9	-0.038	232.5	165452.0	0.636	142.5	88902.0
		CTGAN	0.962	17.1	6434.6	0.028	240.4	154382.5	0.701	124.7	73163.3
SVR	Original data		0.093	167.5	155758.5	-0.077	180.3	164442.4	-0.080	273.1	264198.9
	Original and generated data	bootstrapping	0.097	164.1	152735.4	0.065	171.6	158067.6	-0.079	276.4	263880.2
		SMOTE	0.112	166.6	152330.4	-0.082	185.0	160816.2	-0.063	271.1	260029.3
		GMM	-0.241	201.7	209895.9	-0.249	201.6	209855.8	-0.438	327.4	351701.9
		CTGAN	-0.238	201.5	209458.6	-0.247	201.6	209582.1	-0.434	326.9	350740.5

Table S4. Evaluation metrics of the multi-output RF model with eight features using original and generated data.

Model	Training set (mean)			Validating set (mean)			Test set (mean)		
Metrics	R^2	MAE	MSE	R^2	MAE	MSE	R^2	MAE	MSE
With original data	0.894	42.0	9858.6	0.638	87.2	39279.3	0.817	77.0	28203.3
With original & generated data	0.965	18.4	4327.6	0.902	32.1	12365.2	0.809	72.0	33616.1
